# Hepatoprotective potential of JHB, a functional beverage, in a mouse model of acute alcohol-induced liver injury

**DOI:** 10.3389/fnut.2026.1802918

**Published:** 2026-04-24

**Authors:** Ruizhe Jiang, Yuan Zhu, Yunliang Lei, Xiuwen Jia, Xiaofei Liu, Rathna Silviya Lodi, Xiaoguang Zhan, Tao Ji, Yanlin Zhang, Ting Wang, Lizeng Peng, Yuwei Wang

**Affiliations:** 1 Shaanxi University of Chinese Medicine, Xianyang, Shaanxi, China; 2 Xi’an Medical University, Xi’an, China; 3 Key Laboratory of Agro-Products Processing Technology of Shandong Province, Key Laboratory of Novel Food Resources Processing Ministry of Agriculture, Institute of Food and Nutrition Science and Technology, Shandong Academy of Agricultural Sciences, Jinan, China; 4 Suqian Asia Food Tech Co., Ltd., Suqian, China

**Keywords:** alcohol catabolism, composite plant beverage, flavonoids, hepatoprotection, UPLC-MS/MS

## Abstract

**Purpose:**

Excessive alcohol consumption is a major cause of liver injury worldwide. This study aimed to investigate the protective effects of the plant-based drink Jiuxiangfeng Houwuyou Beverage (JHB) against alcohol-induced acute liver injury in mice.

**Methods:**

The chemical constituents of JHB were characterized using UPLC–MS/MS. Mice were pretreated with JHB for 15 consecutive days and subsequently subjected to an acute ethanol challenge. Behavioral assessments were performed, followed by the collection of blood and tissue samples to evaluate liver function parameters, oxidative stress markers, inflammatory mediators, and the activities of ethanol-metabolizing enzymes. Histopathological examination of liver tissues was also conducted.

**Results:**

Twenty flavonoid compounds in JHB were identified by UPLC–MS/MS analysis. High-level JHB significantly decreased serum ALT and AST levels, MDA content, and liver index, while markedly increasing the activities of ADH, ALDH, and SOD. Histopathological analysis further revealed a level-dependent alleviation of ethanol-induced hepatic injury.

**Conclusion:**

JHB exerts significant protective effects against hepatic injury induced by acute alcohol-induced liver injury, potentially by promoting ethanol metabolism and attenuating oxidative stress and inflammatory responses. Collectively, these results underscore the promise of JHB as a functional plant-based beverage for mitigating alcohol-related liver damage.

## 1 Introduction

Alcohol consumption has increased markedly in recent years and has been accompanied by a growing burden of alcohol-related liver injury. According to the World Health Organization’s *Global Status Report on Alcohol and Health and Treatment of Substance Use Disorders (2024)*, alcohol consumption was associated with approximately 2.6 million deaths globally in 2019. Numerous studies have previously reported that the global annual per capita consumption of pure alcohol increased from 5.9 to 6.5 L between 1990 and 2017, and is projected to reach 7.6 L by 2030, representing an increase of approximately 17% over the next decade (1, 2). Existing evidence has demonstrated particularly that there is no safe level of alcohol consumption (3). Excessive alcohol intake has been proven to trigger a variety of severe diseases, with the damage it inflicts on the body mainly manifested in the following aspects: liver damage (4), neurological damage (5), increased risk of cardiovascular diseases (6), increased risk of cancer (7), pancreatic damage (8), immune suppression (9) and other aspects. Notably, alcohol has been identified as the second largest driver of the increase in liver cancer deaths between 2010 and 2019 (10).

The liver, as the central organ for metabolism and detoxification, eliminates endogenous and exogenous compounds to maintain systemic homeostasis. Ethanol, primarily metabolized hepatically, is largely cleared via oxidation (90–95%), with minimal excretion in breath, urine, or sweat, rendering the liver highly susceptible to alcohol-induced injury (11, 12). Chronic or excessive alcohol consumption is a key risk factor for alcoholic liver disease (ALD) (13). In the liver, ethanol is primarily metabolized into acetaldehyde, a toxic intermediate, via oxidative pathways. The core metabolic enzymes involved include: cytosolic alcohol dehydrogenase (ADH), endoplasmic reticulum/mitochondrial cytochrome P450 2E1 (CYP2E1), and catalase (CAT) (14). Under physiological conditions, ethanol is metabolized to acetaldehyde by ADH in the cytosol, with the major contributing isozymes being ADH1A, ADH1B, ADH1C (Class I) and ADH4 (Class II) (15–17). It should be specifically noted that ADH5 has been reported to participate in ethanol metabolism under relatively high ethanol concentrations, particularly at levels exceeding the metabolic capacity of classical ADH isozymes. Alcohol dehydrogenase 5 (ADH5), also known as class III alcohol dehydrogenase, exhibits an extremely high Km for ethanol (>1 M), rendering it largely inactive under physiological ethanol concentrations. However, it functions as S-nitrosoglutathione reductase (GSNOR), thereby playing a critical role in regulating nitric oxide signaling and glutathione-dependent redox homeostasis (18). Chronic alcohol intake markedly enhances the metabolic involvement of CYP2E1, which is accompanied by excessive generation of reactive oxygen species (ROS) (19). Research indicates that oxidative stress is a fundamental factor in the development of alcoholic liver damage (20). The liver’s susceptibility to alcohol-induced damage stems from the metabolic process itself. During heavy alcohol consumption, the rate of acetaldehyde production exceeds the metabolic capacity of aldehyde dehydrogenase (ALDH), resulting in its accumulation (21). The accumulated acetaldehyde forms covalent adducts with liver proteins/DNA, directly impairing the function of these macromolecules (22). ROS attack membrane lipids, triggering lipid peroxidation chain reactions and generating secondary toxic aldehydes (23). Exposure to acetaldehyde triggers inflammatory reactions in HepG2 cells, leading to the release of pro-inflammatory cytokines such as tumor necrosis factor-*α* (TNF-α) and interleukin-1β (IL-1β) (24). Meanwhile, acetaldehyde directly activates inflammatory pathways such as NF-κB (24, 25), this forms a vicious cycle.

Currently, numerous studies have demonstrated that plants or their active substances can act on alcohol catabolism, liver protection, or antioxidant processes. For example, *Lycium ruthenicum* ameliorates alcohol-related liver injury through modulation of the Nrf2/HO-1/NF-κB signaling pathway, thereby reducing oxidative stress, upregulating Nrf2 and HO-1 expression, and downregulating NF-κB and TNF-*α*, ultimately contributing to the attenuation of ALD (20). Acidic polysaccharides from yam mitigate acute alcoholic liver damage by decreasing inflammation and oxidative stress, preserving intestinal microbiota balance, influencing the liver–intestinal axis, and adjusting the AMPK/PPAR pathway (26). Furthermore, lentinan has demonstrated the ability to maintain liver function, reduce liver fat accumulation, and inhibit oxidative stress and inflammation by encouraging the growth of beneficial gut bacteria in a mouse model of acute alcoholic liver damage (27). Silymarin, a flavonoid derived from *Silybum marianum*, exerted hepatoprotective effects mainly by attenuating oxidative stress and lipid peroxidation (28). Following anthocyanin treatment, the abundance of IFN-*γ*, TNF-*α*, TLR-4, VCAM-1, and CXCL-1 in ALD mice was significantly reduced, indicating that anthocyanins inhibit inflammation by downregulating pro-inflammatory cytokines (29). Studies have shown that extracts from flavonoid-rich fruits such as citrus fruits, mulberries, apples, and jujubes can activate Nrf2-mediated antioxidant responses and exhibit hepatoprotective potential against alcohol-induced liver injury (30). Notably, *Bidens pilosa*, a medicinal and edible plant rich in flavonoids and polyacetylenes, has recently attracted attention for its protective effects against alcohol-induced organ injury. Studies have shown that it alleviates alcohol-induced hepatic steatosis and oxidative stress by modulating lipid metabolism and antioxidant pathways (31). In addition, *B. pilosa* leaves mitigate alcohol-induced chronic kidney injury through antioxidant and anti-inflammatory mechanisms, indicating a broader potential in attenuating alcohol-related multi-organ damage (32). Moreover, *B. pilosa* demonstrates anti-cancer activity under alcohol-related pathological conditions, including inhibition of colon cancer progression via regulation of inflammation- and apoptosis-related pathways (33). However, previous studies have lacked exploration into the combined anti-ALD effects of multiple medicinal and edible homologous substances. Therefore, the treatment of ALD through pharmaceutical approaches has become an urgent need, and meanwhile, previous studies have provided a direction for our research on the anti-alcohol and hepatoprotective effects of natural products derived from multiple plants possessing both therapeutic and nutritional functions.

To explore the potentiality of multiple plants possessing both therapeutic and nutritional functions, we have developed a compound plant beverage named Jiuxiangfeng Houwuyou Beverage (JHB). Its main ingredients include hawthorn juice, apple juice, crystalline fructose, *Ampelopsis grossedentata* leaf extract, pueraria powder, sodium hyaluronate, and food additives. The main medicinal and edible homologous plants used in JHB have been previously confirmed to have significant anti-ALD effects. Kim et al. (34) suggested that hawthorn possesses hepatocyte-protective, anti-adipogenic, anti-cancer, anti-inflammatory, and anti-fibrotic effects. Apple juice has been confirmed to possess significant anti-ALD activity after fermentation with *Lactobacillus* CICC6064 (35). The extract of *Ampelopsis grossedentata* leaves exerts its hepatoprotective effect by reducing lipid peroxidation and enhancing antioxidant activity (36). Pueraria extract can delay the progression of ALD by regulating oxidative stress and inflammatory responses (37). Curcumin, the main bioactive compound in medicinal foods such as *Curcuma longa* rhizome, exerts antioxidative, anti-inflammatory, and pro- or anti-apoptotic effects, contributing to its therapeutic potential in various diseases (38). However, previous studies have lacked empirical evidence on the hepatoprotective effects of multi-component medicinal and edible homologous beverages in ALD. In this study, JHB was developed and evaluated in a mouse model of acute alcohol-induced liver injury. The present study reports that pretreatment with multiple plants possessing is associated with changes in ethanol metabolism and oxidative stress markers, providing preliminary evidence of potential hepatoprotective effects.

## 2 Materials and methods

### 2.1 Materials and reagents

The compound plant beverage JHB was provided by the Shandong Academy of Agricultural Sciences. Baijiu (52% alcohol by volume, vol) was purchased from Beijing Red Star Co., Ltd. (Beijing, China). Silymarin (Purity ≥ 80%; cat. no. S304299-5 g), used as the positive control sample, was acquired from Aladdin Reagent Co., Ltd. (Shanghai, China). ELISA kits for TNF-*α* (catalog number EK0527), IL-1β (catalog number EK0394), IL-6 (catalog number EK0411), and IL-4 (catalog number EK0405) were obtained from BOSTER in Wuhan, China. Kits for ALT (cat. no. C009-2-1), AST (cat. no. C010-2-1), T-SOD (cat. no. A001-3-2), catalase (CAT; cat. no. A007-1-1), GSH (cat. no. A006-2-1), MDA (cat. no. A003-1-2), ADH (cat. no. A083-1-1), and ALDH (cat. no. A075-1-1) were all obtained from Nanjing Jiancheng Bioengineering Institute (Nanjing, China).

### 2.2 UPLC-MS/MS analysis

A Shimadzu Nexera X2 LC-30 AD high-performance liquid chromatography system (Shimadzu, Japan) with an ACQUITY UPLC HSS T3 column (1.8 μm, 2.1 × 50 mm; Waters, USA) was used for chromatographic separation. Consisting of solvent A (0.1% formic acid in water) and solvent B (methanol), the mobile phase was used. Samples were kept at 4 °C in the autosampler, and the column was maintained at 40 °C. A flow rate of 0.25 mL/min was used, with an injection volume of 2 μL. The gradient elution sequence was: 0–1 min, 5% B; 1–2 min, 5–40% B; 2–8 min, 40–50% B; 8–15 min, 50–100% B; 15–16 min, 100% B; 16–16.1 min, 100–5% B; and 16.1–18 min, 5% B. A QTRAP 6500 + mass spectrometer (AB SCIEX, USA) with an electrospray ionization (ESI) source was used for mass spectrometric detection, functioning in both positive and negative ion modes. The ESI parameters in positive ion mode included a source temperature of 500 °C, GAS1 at 55, GAS2 at 60, CUR at 35, and an ISVF of +5,500 V. The settings for negative ion mode were: source temperature at 500 °C, GAS1 at 55, GAS2 at 60, CUR at 35, and ISVF at −4,500 V. Multiple reaction monitoring (MRM) mode was utilized for the quantification of target ions.

### 2.3 Mouse model for acute alcohol-induced hepatic injury

Thirty-six male Kunming mice, specific pathogen-free and weighing 30–35 grams, aged 6–8 weeks, were acquired from Chengdu Dashuo Laboratory Animal Co., Ltd. in Sichuan Province, China. All mice used in the experiments were housed in cages at the animal facility, where conditions were controlled: temperature maintained at 20–25 °C, relative humidity at 50–60%, and a 12-h light–dark cycle (illumination from 8:00 to 20:00), with ad libitum access to water and food. All experimental animals were treated in a humane manner. This study was performed in compliance with the regulations of the Ethics Committee of Shaanxi University of Chinese Medicine (No. SUCMDL20250728001).

### 2.4 Animal experiment

After 3 days of acclimatization, a total of 36 mice were randomly allocated into six groups (*n* = 6 per group): (1) control group; (2) model group; (3) positive control group; (4) low-dose JHB group (5 mL/kg); (5) medium-dose JHB group (7.5 mL/kg); and (6) high-dose JHB group (15 mL/kg). Throughout the experiment, mice in both the control, control and model groups were given a daily intragastric dose of 0.9% saline solution at a rate of 10 mL/Kg. Meanwhile, the positive control group was administered 200 mg/kg per day by oral gavage, while the three JHB groups received oral gavage at doses of 5, 7.5, and 15 mL/kg, respectively. Each animal was leveled once daily, with the timing of intragastric administration documented. This intervention period lasted for a period of 15 days, during which body weights were recorded daily; subsequent dosages were adjusted proportionally based on body weight fluctuations. Thirty minutes after the final administration, all groups except the control group, received normal saline, received white liquor at 12 mL/kg. At this point, the intragastric dosing regimen was terminated. For the mice receiving Baijiu on day 15, the performance in the climbing test, pole test, and balance beam test was recorded. Subsequently, the animals were euthanized, and liver tissue aliquots were harvested from each mouse.

### 2.5 Climbing test in mice

To assess limb muscle strength and coordination in experimental animals, a climbing test was performed (39). After administering alcohol, immediately place the mouse in the center of a 40 cm long, 25 cm wide, and 1 cm spaced horizontal suspended metal net. Immediately flip the metal net to make the mouse upside down. Recording of the climbing time for each group was performed. Termination of the experiment occurred when a mouse fell three times, and the mean climbing time was calculated.

### 2.6 Balance beam test in mice

To assess the coordination and balance abilities of experimental animals during fine motor tasks, the mice balance beam test was performed (40). During the test, animals were required to stand upright and traverse a narrow beam to reach a safety box. Briefly, a wooden stick (60 cm in length and 1.5 cm in width) was placed horizontally at a height of 50 cm above the ground. A desk lamp was positioned at the starting end of the beam, and a dark box was placed at the opposite end to create a light–dark environment. During the experiment, mice were placed at the illuminated starting end, and the time required to traverse the beam and enter the dark box was recorded. Each mouse was measured three times per day with a 10-min interval between trials. The first 2 days were used for training, and from the third day onward, the balance beam traversal time was recorded for analysis.

### 2.7 Pole-climbing test in mice

To evaluate motor function in experimental animals, including limb coordination and grip strength, the mouse pole-climbing test was performed. Shorter descent times indicated improved motor performance, whereas longer times reflected motor impairment. The apparatus consisted of a wooden pole (measuring 2 cm in diameter and 47 cm in length) wrapped with gauze to increase friction. Prior to testing, mice were trained to descend the pole using only their hindlimbs; animals that remained stationary or climbed upward were excluded. During the formal experiment, mice were administered white liquor by intragastric gavage. Placement of each mouse at the top of the pole, with the head facing downward, was performed 10 min after gavage, and the time required to descend the pole was recorded. Each mouse was subjected to three tests, and the mean of these was used for further analysis.

### 2.8 The liver, spleen and kidney indices of mice

The mice were euthanized through cervical dislocation, and their liver, spleen, and kidneys were quickly collected and kept on ice. Each organ was thoroughly rinsed with pre-cooled physiological saline, and surface moisture was blotted dry with filter paper. The liver index was calculated by taking the ratio of liver weight to body weight, with organ weights being recorded. Changes in this index, whether increases or decreases, may reflect alterations in liver morphology, including swelling, congestion, hypertrophy, atrophy, or other degenerative processes (41). The liver index was calculated according to Equation (1). The spleen index was measured as the proportion of spleen weight to body weight. An elevation in this index indicates potential splenic swelling, congestion, or hypertrophy, while a reduction suggests possible splenic atrophy or other degenerative changes. The spleen index was computed using Equation (2). The kidney index was established as the ratio of kidney weight to body weight. An increase in this index indicates potential renal swelling, congestion, or hypertrophy, whereas a decrease suggests possible renal atrophy or other degenerative changes. The kidney index was calculated in accordance with Equation (3).


$$\text{Liver index}=\frac{\text{Liver quality}}{\text{Body weight}}\times 100\%$$ (1)

$$\text{Spleen index}=\frac{\text{Spleen quality}}{\text{Body weight}}\times 100\%$$ (2)

$$\text{Kidney index}=\frac{\text{Kidney quality}}{\text{Body weight}}\times 100\%$$ (3)

### 2.9 Assessment of hematological parameters in mice

Three hours following the oral administration of white liquor, blood was drawn from the retro-orbital sinus of the mice. The serum was separated by centrifugation at 9000 rpm for 10 min at 4 °C. The activities of ALT, AST, TNF-*α*, IL-1β, IL-4, IL-6, SOD, CAT, GSH, MDA, and ADH in the serum were quantified using commercial assay kits, with operations conducted following the manufacturers’ instructions. Measurements were performed using a 318 M microplate reader (Shanghai Lichen Analytical Instrument Co., Ltd., Shanghai, China).

### 2.10 Determination of hepatic parameters in mice

Following euthanasia, liver tissue was homogenized in physiological saline at a 1:9 (w/v) ratio to prepare liver homogenates. The homogenates underwent centrifugation at 12,000 rpm for 10 min at 4 °C, and the supernatants were then collected. Commercial assay kits were used to determine hepatic ALDH doses according to the manufacturers’ instructions, and absorbance was measured with a 318 M microplate reader (Shanghai Lichen Analytical Instrument Co., Ltd., Shanghai, China).

### 2.11 Histopathological analysis of liver

Hematoxylin–eosin (H&E) staining was used to assess liver morphology. Rinsing of liver tissues was performed using ice-cold phosphate-buffered saline (PBS), sectioned into 5-μm-thick slices, fixed in 4% paraformaldehyde, and subjected to histopathological analysis (42). Images were acquired using a light microscope.

### 2.12 Data statistics and analysis

Data are presented as the mean ± standard deviation (SD). One-way analysis of variance (ANOVA) was used for group comparisons, followed by Tukey’s post-hoc test with Holm-Bonferroni correction for multiple comparisons. Statistical significance was defined as *p* < 0.05 and extreme significance as *p* < 0.01. All statistical analyses were performed using GraphPad Prism 8 (GraphPad Software, San Diego, CA, USA), and all figures were prepared using Adobe Illustrator 2021.

## 3 Results

### 3.1 UPLC-MS/MS-based identification and quantification of flavonoids in JHB

**Figure 1:**
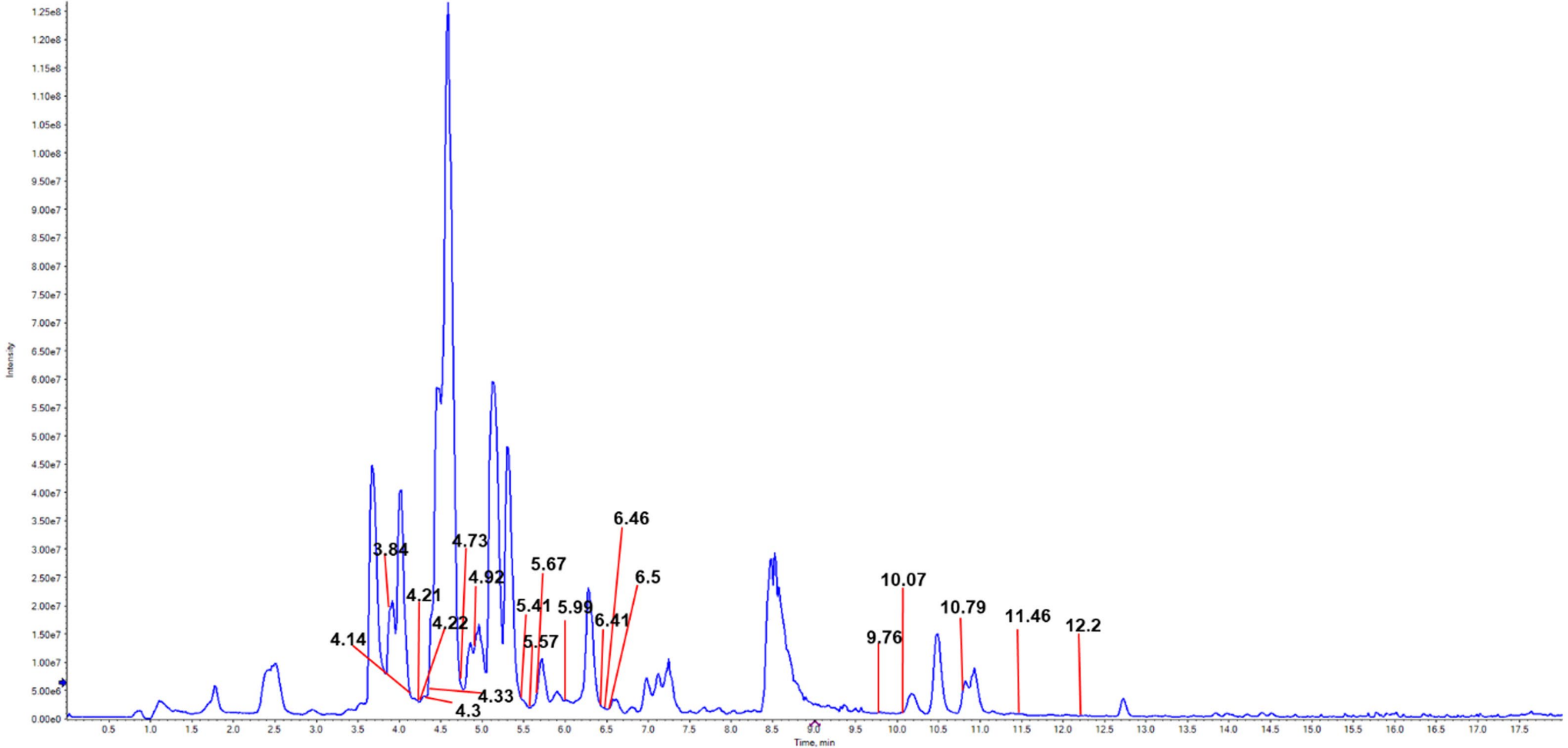
UPLC-MS/MS total ion chromatogram of JHB.

Flavonoid structures in JHB were tentatively identified according to the molecular weights and retention times of protonated ions, along with identification through database matching and authentic reference standards, resulting in the identification of 20 compounds (Figure 1; Table 1). These include Catechin (RT 3.84), Mirificin (RT 4.14), L-Epicatechin (RT 4.21), Puerarin (RT 4.22), 3′-Methoxypuerarin (RT 4.30), Dihydromyricetin (RT 4.33), Daidzin (RT 4.73), Glycitin (RT 4.92), Taxifolin (RT 5.41), Vitexin (RT 5.57), Genistin (RT 5.67), Isovitexin (RT 5.99), Rutin (RT 6.41), Hesperidin (RT 6.46), Quercetin 3-glucoside (RT 6.50), Glycitein (RT 9.76), 7,4’-Dihydroxyflavone (RT 10.07), Genistein (RT 10.79), Norwogonin (RT 11.46), and Formononetin (RT 12.20). Among these, Puerarin, 3′-Methoxypuerarin, Dihydromyricetin, Glycitin, Taxifolin, Genistin, Rutin, Quercetin 3-glucoside, Glycitein, and 7,4’-Dihydroxyflavone were present at higher doses, and are likely responsible for the alcohol-metabolizing and hepatoprotective effects of JHB.

**Table 1:** Analysis of flavonoid constituents in JHB via UPLC-MS/MS.

No	RT (min)	Compounds	Formula	Molecular weight	Concentration (nmol/mL)
1	3.84	Catechin	C_15_H_14_O_6_	290.27	11.29
2	4.14	Mirificin	C_26_H_28_O_13_	548.5	5.68
3	4.21	Epicatechin	C_15_H_14_O_6_	290.27	4.11
4	4.22	Puerarin	C_21_H_20_O_9_	416.4	36.86
5	4.3	3′-Methoxypuerarin	C_22_H_22_O_10_	446.4	28.35
6	4.33	Dihydromyricetin	C_15_H_12_O_8_	320.25	1470.36
7	4.73	Daidzin	C_21_H_20_O_9_	416.4	11.72
8	4.92	Glycitin	C_22_H_22_O_10_	446.4	24.94
9	5.41	Taxifolin	C_15_H_12_O_7_	304.25	51.81
10	5.57	Vitexin	C_21_H_20_O_10_	432.4	12.35
11	5.67	Genistin	C_21_H_20_O_10_	432.4	27.71
12	5.99	Isovitexin	C_21_H_20_O_10_	432.4	4.84
13	6.41	Rutin	C_27_H_30_O_16_	610.5	37.45
14	6.46	Hesperidin	C_28_H_34_O_15_	610.6	3.07
15	6.5	Quercetin 3-glucoside	C_21_H_20_O_12_	464.4	141.05
16	9.76	Glycitein	C_16_H_12_O_5_	284.26	139.42
17	10.07	7,4’-Dihydroxyflavone	C_15_H_10_O_4_	254.24	21.88
18	10.79	Genistein	C_15_H_10_O_5_	270.24	5.62
19	11.46	Norwogonin	C_15_H_10_O_5_	270.24	11.83
20	12.2	Formononetin	C_16_H_12_O_4_	268.26	9.57

### 3.2 The impact of JHB on the behavior of mice

**Figure 2:**
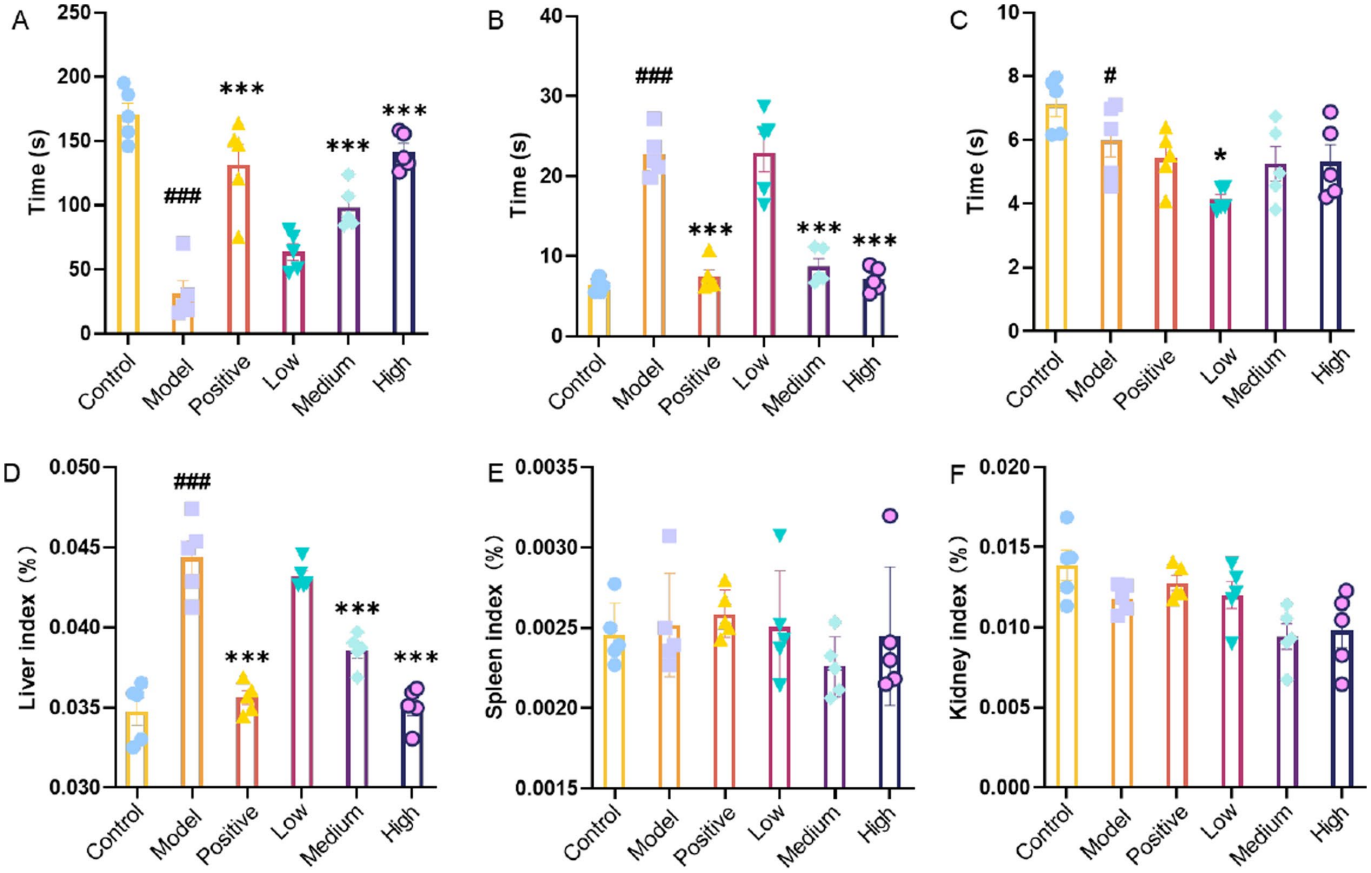
Impact of JHB on the behavior of mice, as well as the influence of JHB on the indicators of mice’s liver, spleen and kidneys. **(A)** Mouse climbing experiment. **(B)** Mouse balance beam experiment. **(C)** Mouse climbing pole experiment. **(D)** Liver index. **(E)** Spleen index. **(F)** Kidney index. Values significantly different from the control are indicated by hash signs (^#^*p* < 0.005, ^###^*p* < 0.001). Values significantly different from the ethanol are indicated by asterisks (^*^p < 0.005, ^***^p < 0.001). Control: control group; Model: model group; Positive: silymarin group; Low: JHB low-dose group; Medium: JHB medium-dose group; High: JHB high-dose group.

To assess the effects of alcohol and different doses of JHB on motor coordination, balance, and neuromuscular function, climbing, balance beam, and pole-climbing tests were conducted. Compared to the control group, the model group had a significantly impaired performance in the climbing test (Figure 2A) (*p* < 0.001). Relative to the model group, there was a significant boost in climbing performance in the positive control and JHB medium- and high-dose groups (*p* < 0.001), with the medium- and high-dose groups showing the greatest gains. In the balance beam test (Figure 2B), a significant decrease in performance was observed in the model group relative to the control group (*p* < 0.001). Compared with the model group, performance was significantly improved in the positive control and in the JHB medium- and high-dose groups (*p* < 0.001), with more pronounced effects in the medium- and high-dose groups. Compared with the control group, the model group (*p* < 0.05) exhibited significantly impaired performance in the pole-climbing test (Figure 2C). Mice in the positive control and JHB low-dose groups exhibited significant improvements relative to the model group (*p* < 0.05).

### 3.3 Impact of JHB on hepatic, splenic, and renal indices in mice

During the experiment, all groups experienced a gradual increase in body weight, though the gain was slight. No major changes in body weight were detected among the groups, suggesting that intragastric JHB treatment did not have a substantial influence. The liver index, a useful parameter for assessing liver pathology such as hepatomegaly or atrophy, is known to increase in mice following acute alcohol exposure due to hepatic hypertrophy. To evaluate the effects of alcohol and varying doses of JHB on organ health, liver, spleen, and kidney indices were measured. In Figure 2D, a substantial elevation in the liver index is evident in the model group as opposed to the control group (*p* < 0.001). Compared to the model group, the liver index was significantly more reduced in the positive control group and the medium- and high-dose JHB groups (*p* < 0.001), with the medium- and high-dose JHB groups showing more notable reductions. As shown in Figure 2E, no statistically significant differences in spleen index were observed among the groups; as shown in Figure 2F, no statistically significant differences in kidney index were observed among the groups, indicating that JHB did not exert a significant effect on the spleen or kidney.

### 3.4 The effect of JHB on oxidative stress in mice

**Figure 3:**
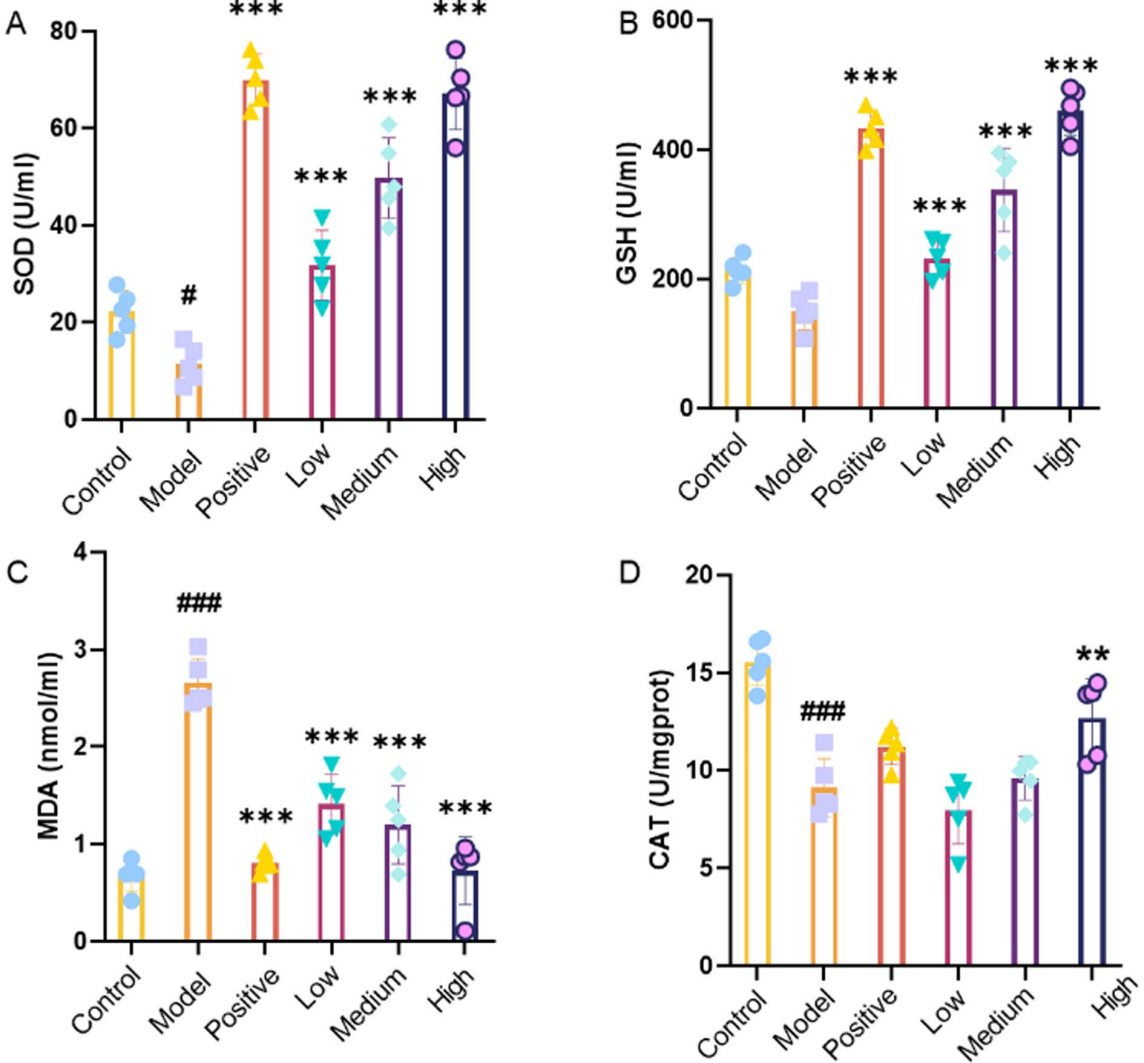
The effects of JHB on oxidative stress in mice. **(A)** SOD; **(B)** GSH; **(C)** MDA; **(D)** CAT. Values significantly different from the control are indicated by hash signs (^#^*p* < 0.005, ^###^*p* < 0.001). Values significantly different from the ethanol are indicated by asterisks (^**^p < 0.005, ^***^p < 0.001). Control: control group; Model: model group; Positive: silymarin group; Low: JHB low-dose group; Medium: JHB medium-dose group; High: JHB high-dose group.

The effects of alcohol and JHB at different doses on serum oxidative stress measurement of SOD, GSH, MDA, and CAT activities was performed to further evaluate the samples. According to Figure 3A, SOD activity significantly decreased in the model group versus the control group (*p* < 0.05). Compared with the model group, SOD activity was significantly increased in the positive control group and in the JHB low-, medium-, and high-dose groups (*p* < 0.001), with more pronounced increases observed in the medium- and high-dose JHB groups. Figure 3B illustrates that GSH doses were markedly higher in the positive control group and all JHB-treated groups compared to the model group (*p* < 0.001). Compared to the low-dose group, the medium- and high-dose JHB groups experienced a greater rise in GSH doses. According to Figure 3C, the model group exhibited a significant rise in MDA content compared to the control group (*p* < 0.001). In contrast, MDA doses were significantly reduced in the positive control group and in the JHB low-, medium-, and high-dose groups compared with the model group (*p* < 0.001), with more pronounced reductions seen in the medium- and high-dose groups. As shown in Figure 3D, CAT activity was significantly lower in the model group than in the control group (*p* < 0.001). CAT activity was significantly increased in the JHB high-dose groups compared to the model group (*p* < 0.005), with the medium- and high-dose JHB groups showing a more pronounced rise.

### 3.5 Impact of JHB on inflammatory mediators in mice

**Figure 4:**
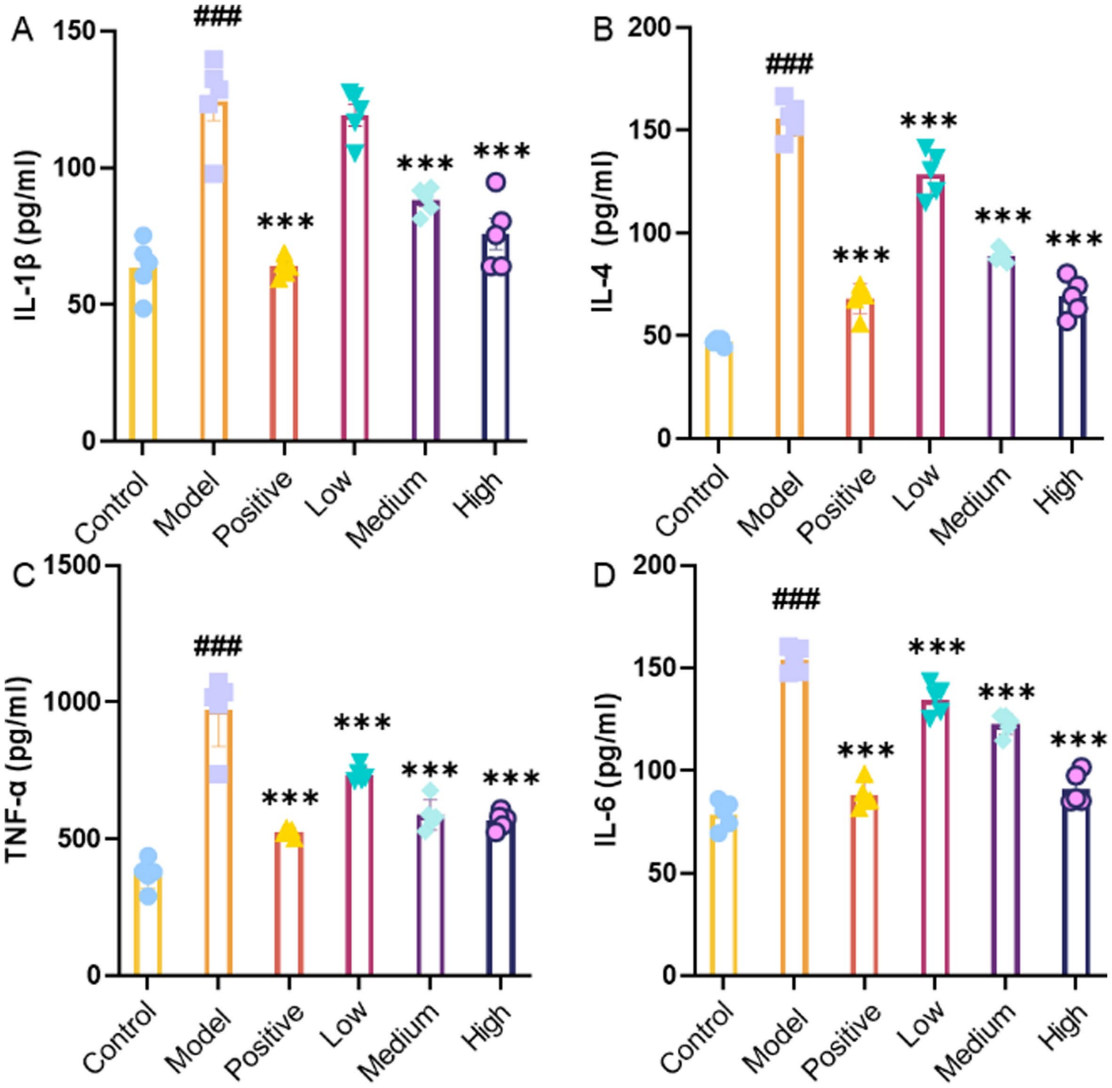
The effects of JHB on inflammatory factors in mice. **(A)** IL-1β; **(B)** IL-4; **(C)** TNF-*α*; **(D)** IL-6. Values significantly different from the control are indicated by hash signs (^###^*p* < 0.001). Values significantly different from the ethanol are indicated by asterisks (^***^p < 0.001). Control: control group; Model: model group; Positive: silymarin group; Low: JHB low-dose group; Medium: JHB medium-dose group; High: JHB high-dose group.

To further assess the impact of alcohol exposure and JHB treatment at various doses on liver inflammation, the concentrations of IL-1β, IL-4, TNF-*α*, and IL-6 were measured. As shown in Figure 4, in comparison to the control group, the levels of IL-1β, IL-4, TNF-α, and IL-6 in the model group were significantly higher following acute alcohol exposure (*p* < 0.001), indicating that acute alcohol challenge induced a marked inflammatory response in the liver. According to Figure 4A, IL-1β levels significantly decreased in the positive control group and the medium- and high-dose JHB groups relative to the model group (*p* < 0.001). Figure 4B illustrates that IL-4 levels were notably reduced in both the positive control group and all groups treated with JHB compared to the model group (*p* < 0.001). Figure 4C illustrates that TNF-α levels were markedly reduced in all JHB level groups compared to the model group (*p* < 0.001). Similarly, Figure 4D illustrates that IL-6 levels were markedly lower in both the positive control group and all groups treated with JHB compared to the model group (*p* < 0.001). Taken together, these results demonstrate that JHB intervention effectively attenuated alcohol-induced inflammation, with more pronounced anti-inflammatory effects observed at medium and high levels.

### 3.6 Effects of JHB on ADH and ALDH activities in mice

**Figure 5:**
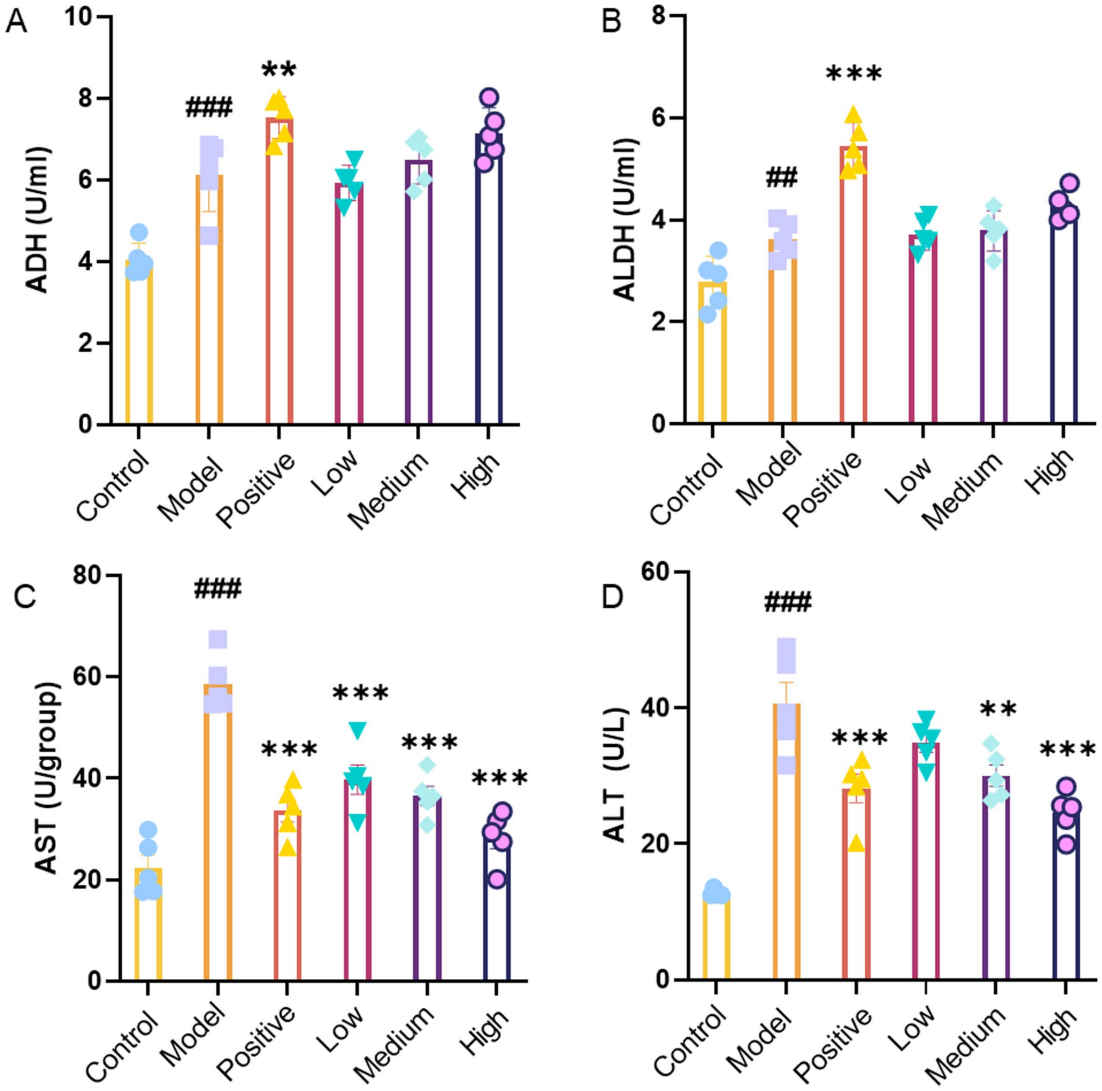
The effects of JHB on the enzymes related to alcohol metabolism in mice. **(A)** ADH; **(B)** ALDH; **(C)** AST; **(D)** ALT. Values significantly different from the control are indicated by hash signs (^##^*p* < 0.005, ^###^*p* < 0.001). Values significantly different from the ethanol are indicated by asterisks (^**^p < 0.005, ^***^p < 0.001). Control: control group; Model: model group; Positive: silymarin group; Low: JHB low-dose group; Medium: JHB medium-dose group; High: JHB high-dose group.

To further evaluate the effects of alcohol exposure and JHB treatment at different levels, serum ADH activity and hepatic ALDH activity were measured. As shown in Figure 5A, ADH activity in the model group was significantly increased compared with that in the control group (*p* < 0.001), indicating activation of hepatic alcohol metabolism following acute alcohol exposure. In addition, ADH activity showed a gradual increase with increasing levels of JHB. According to Figure 5B, there was a marked increase in ALDH activity in the model group when contrasted with the control group (*p* < 0.05). Compared to the model group, the positive control group exhibited a notable increase in ALDH activity (*p* < 0.001). Similarly, ALDH activity exhibited an increasing trend with increasing levels of JHB. Taken together, these results indicate that JHB intervention enhanced hepatic alcohol-metabolizing enzyme activities, suggesting an improvement in hepatic alcohol metabolic capacity in mice subjected to acute alcohol exposure.

### 3.7 Effects of JHB on serum ALT and AST levels in mice

The effects of alcohol exposure and JHB treatment at different levels on hepatic injury were further evaluated by measuring ALT and AST activities. Figure 5C illustrates that the model group had a significantly higher ALT activity compared to the control group (*p* < 0.001). In comparison to the model group, the positive control group and all JHB level groups showed a significant decrease in ALT activity (*p* < 0.001). In the same way, AST activity was considerably higher in the model group than in the control group (*p* < 0.001).Figure 5D illustrates that AST activity was notably reduced in the positive control group (*p* < 0.001), the medium-level JHB group (*p* < 0.005), and the high-level JHB group (*p* < 0.001) compared to the model group. Taken together, these results indicate that JHB treatment effectively alleviated alcohol-induced hepatic injury, with more pronounced protective effects observed at medium and high levels.

### 3.8 Histopathological analysis of liver tissue

**Figure 6:**
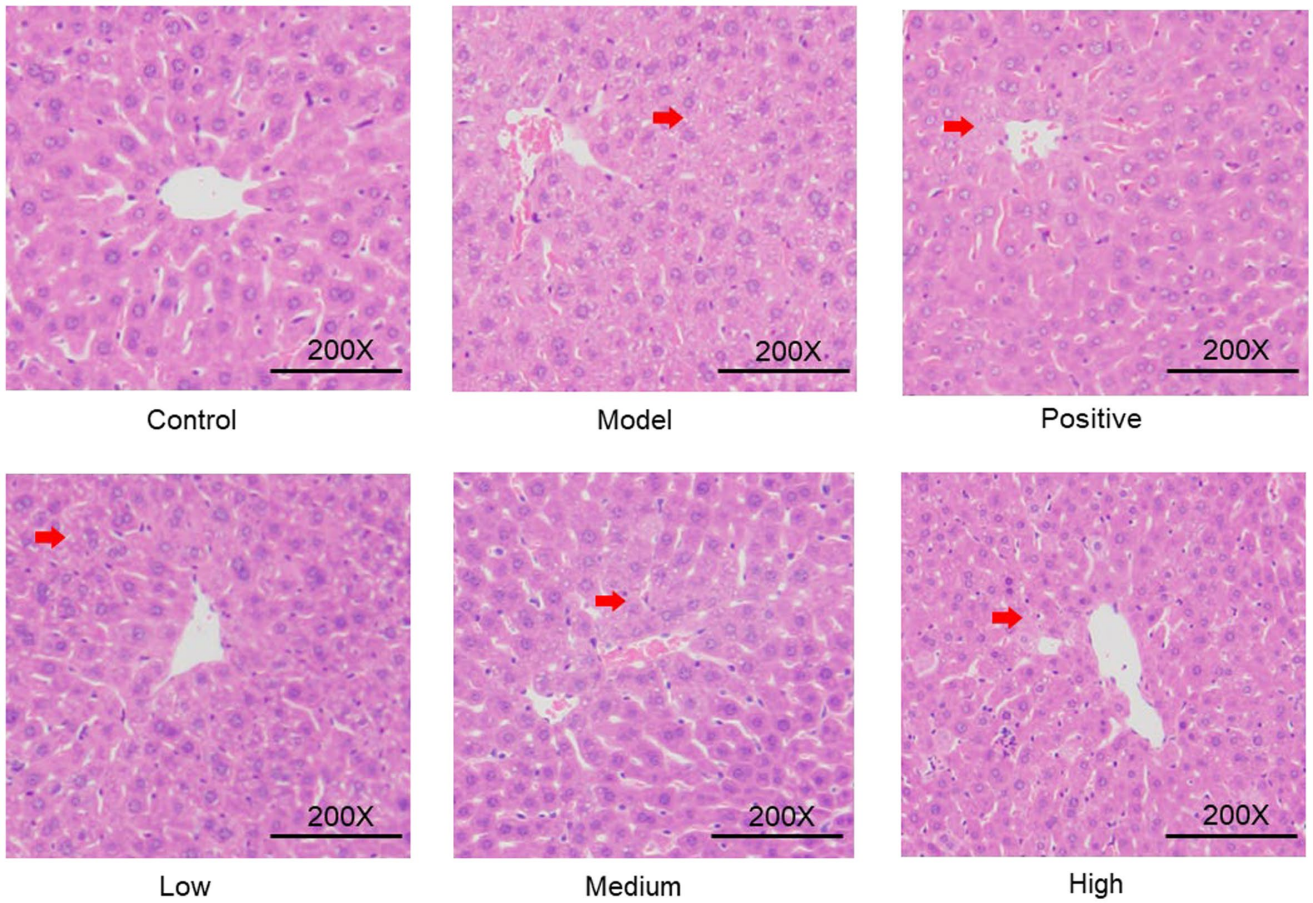
Effects of JHB on pathological changes in mouse liver.

The effects of alcohol and JHB at different levels on the liver were further evaluated via hematoxylin–eosin (H&E) staining. As presented in Figure 6, the liver structure of the control group was normal. Hepatocytes were arranged in a single-layer plate-like radial pattern centered on the central vein; hepatic sinusoids were well dilated; hepatocytes exhibited a polygonal shape with round nuclei; and the nuclear-to-cytoplasmic ratio ranged from 1:4 to 1:6. At 3 h after alcohol administration, the liver tissue of the model group still maintained a normal structure. However, hepatocytes in the central region of the hepatic lobules showed increased volume, with loose and lightly stained cytoplasm, and a decreased nuclear-to-cytoplasmic ratio. Hepatic sinusoids in this region were compressed, narrowed, or even occluded. The alcohol-induced liver damage was alleviated to a certain extent in the positive control group. Alcohol-induced liver damage was alleviated to varying degrees by JHB at different levels, exhibiting a clear level-response relationship. Among these groups, the degree of cytoplasmic looseness in the liver tissue of the high-level JHB group was similar to that of the positive control group. Therefore, it can be concluded that acute alcohol-induced liver damage was alleviated to a certain extent by JHB.

## 4 Discussion

Recently, as living standards have improved, there has been a notable surge in alcohol consumption in China. Alcohol use is connected with liver damage, which may increase the likelihood of liver fibrosis and hepatocellular carcinoma. Consequently, there is growing interest in functional beverages that can mitigate alcohol-induced liver damage. In this context, we developed a plant-based beverage, JHB, with potential alcohol-detoxifying and hepatoprotective properties. In this study, we assessed the liver-protective effects of JHB in a mouse model of liver damage caused by acute alcohol exposure. Our results demonstrated that acute alcohol exposure significantly disrupted hepatic function, induced oxidative stress, elevated pro-inflammatory cytokines, and activated alcohol-metabolizing enzymes. Intervention with JHB effectively alleviated these alcohol-induced alterations, as evidenced by the normalization of ALT and AST activities, enhancement of antioxidant enzyme activities (SOD and CAT), reduction of lipid peroxidation (MDA), modulation of inflammatory cytokines (IL-1β, IL-4, TNF-*α*, and IL-6), and improvement in ADH and ALDH activities. These findings indicate that JHB possesses an associated with hepatoprotective effects, highlighting its potential as a functional beverage for mitigating acute alcohol-induced liver injury.

Extensive evidence indicates (43, 44) that flavonoids are the major functional components responsible for the alcohol-detoxifying and hepatoprotective effects of plant-derived beverages, exerting antioxidant, anti-inflammatory, and metabolic modulatory actions against alcohol-induced liver injury. For example, quercetin, quercetin-3-glucoside, and rutin have been shown to significantly prevent ethanol-induced hepatotoxicity by decreasing aminotransferase activities and reducing inflammatory responses, in part via activation of antioxidant pathways such as the Nrf2/ARE signaling cascade in liver cells exposed to ethanol (45). More broadly, dietary flavonoids have been reported to enhance alcohol-metabolizing enzyme activities, attenuate oxidative stress, and suppress pro-inflammatory signaling in alcohol-challenged models, supporting their role in protecting the liver from ethanol-induced damage (46). In the present study, UPLC–MS/MS analysis revealed that the primary flavonoid constituents of JHB included catechin, puerarin, 3′-methoxypuerarin, dihydromyricetin, glycitin, taxifolin, genistin, rutin, quercetin-3-glucoside, glycitein, and 7,4′-dihydroxyflavone. The presence of these bioactive flavonoids, many of which have been associated with hepatoprotective effects in previous research, may contribute substantially to the alcohol-detoxifying and liver-protective properties observed in JHB. These findings are consistent with earlier research (47, 48) demonstrating the liver-protective properties of flavonoid-rich compounds against liver damage caused by alcohol. Acute alcohol exposure significantly impaired motor coordination and neuromuscular function, as evidenced by decreased performance in climbing, balance beam, and pole-climbing tests (Figures 2A–C). Behavioral impairments resulting from ethanol toxicity have been linked to oxidative stress and neuroinflammation, and interventions with flavonoid-rich extracts or antioxidants have been shown to attenuate such deficits, improving motor performance in rodent models of alcohol intoxication (49). The behavioral improvements observed with JHB, especially at medium and high levels, are consistent with the neuroprotective effects attributed to plant bioactives in ethanol models. Body weight was observed to gradually increase in all groups, with no significant differences detected, indicating that JHB administration did not adversely affect growth. The liver index was significantly elevated in the model group due to alcohol-induced hepatomegaly, whereas this increase was attenuated in a level-dependent manner following JHB treatment (Figure 2D). In contrast, spleen and kidney indices were not significantly affected (Figures 2E,F), suggesting that the protective effects of JHB were primarily directed toward the liver. It has been reported that alcohol-induced hepatomegaly is largely mediated by oxidative and inflammatory stress, and that flavonoid-containing extracts can selectively mitigate liver enlargement (50). ROS are generated during ethanol metabolism, leading to disruption of the redox balance and contributing to lipid peroxidation and oxidative injury in hepatocytes (51). In the present study, serum SOD, CAT, and GSH activities were found to be decreased, whereas MDA content was increased following alcohol exposure (Figures 3A–D), indicating the occurrence of oxidative damage. These alterations were significantly reversed by JHB treatment, with antioxidant enzyme activities enhanced and MDA levels reduced, suggesting that redox homeostasis was restored. These findings are consistent with emerging evidence that flavonoids may exert hepatoprotective effects and contribute to observed changes in alcoholic liver disease models (52, 53). Alcoholic liver injury is closely associated with activation of innate immune signaling and increased pro-inflammatory cytokines such as IL-1β, TNF-*α*, and IL-6 (54). These cytokines play critical roles in mediating inflammation: IL-1β promotes the recruitment and activation of inflammatory cells, TNF-α contributes to hepatocyte apoptosis and necrosis, and IL-6 modulates the acute-phase response and promotes inflammatory signaling in the liver. Elevated levels of these cytokines are therefore indicative of inflammatory stress and liver injury following acute alcohol exposure. In this study, ethanol exposure significantly increased serum IL-1β, IL-4, TNF-α, and IL-6 levels (Figures 4A–D), indicating the induction of a pronounced inflammatory response. In the present study, IL-4, an anti-inflammatory cytokine, was significantly elevated in the model group compared with the normal group, whereas JHB treatment reduced IL-4 levels relative to the model group. This decrease is consistent with an overall attenuation of inflammatory stress rather than direct suppression of IL-4, as supported by concurrent reductions in pro-inflammatory cytokines, including TNF-α, IL-6, and IL-1β. These results are consistent with previous reports demonstrating that flavonoids and related phytochemicals can mitigate ethanol-induced inflammation by modulating pro- and anti-inflammatory mediators and potentially influencing signaling pathways such as NF-κB (55).

Ethanol is oxidized by ADH to acetaldehyde, which is further metabolized by ALDH to acetate, with both steps critical for detoxification and limiting acetaldehyde toxicity (56). In the present study, JHB significantly enhanced serum ADH and hepatic ALDH activities (Figures 5A,B), suggesting a possible enhancement in ethanol catabolism. These findings are consistent with studies showing that flavonoid components such as puerarin can elevate ADH and ALDH activities and ameliorate hepatic lesions in alcoholic liver injury models (57). Elevated ALT and AST are widely recognized markers of hepatocyte membrane disruption due to ethanol toxicity (58). In this study, alcohol exposure increased ALT and AST activities, while JHB treatment significantly lowered both (Figures 5C,D), indicating preserved hepatocyte integrity. These biochemical improvements align with reports that natural antioxidants reduce serum transaminases in ethanol-induced liver injury by attenuating oxidative and inflammatory damage (59). Taken together, these results suggest that JHB is associated with mitigation of alcohol-induced liver injury, potentially through promotion of ethanol metabolism (via ADH/ALDH), restoration of antioxidant defenses, suppression of inflammatory cytokines, and preservation of hepatocyte structure. The high concentrations of bioactive flavonoids identified, such as puerarin, dihydromyricetin, and related compounds, may contribute to these protective effects, likely via their antioxidative and anti-inflammatory properties. These mechanisms could underlie the observed hepatoprotective effects in alcoholic liver disease models by modulating redox balance, metabolic detoxification, and immune signaling pathways.

However, the present study was limited by its short treatment duration and focus solely on acute alcoholic liver injury. Future investigations using chronic alcohol exposure models are warranted to assess the long-term efficacy of JHB and to further explore the molecular mechanisms underlying its hepatoprotective effects and its association with alcohol-metabolizing enzyme activity. This study is constrained by the absence of a JHB-only group, which prevents evaluation of baseline beverage effects and obscures the distinction between specific phytochemical actions and nonspecific formulation effects. Additional limitations include the use of an acute alcohol exposure model, the lack of direct metabolic measurements, limited pathway analyses, and restricted histological scoring, all of which constrain mechanistic interpretation. Future investigations should incorporate JHB-only groups, measure ethanol, acetaldehyde, and acetate levels, assess CYP2E1 activity, include both sexes, and analyze serum and hepatic markers of oxidative stress, cytokine responses, and enzyme activities. Addressing these limitations will provide more definitive and mechanistic insights into the hepatoprotective efficacy and safety profile of JHB as a functional beverage.

## 5 Conclusion

The present study formulated a plant-based functional beverage, JHB, using hawthorn, apple, grapevine leaves with significant teeth, and kudzu root as raw materials. UPLC–MS/MS analysis identified 20 compounds in JHB, with flavonoids being the major constituents, conferring strong antioxidant activity. In a mouse model of acute alcohol-induced liver injury, JHB exhibited notable hepatoprotective and was associated with increased activities of alcohol-metabolizing enzymes. Administration of JHB enhanced ADH, ALDH, and SOD activities, increased the liver index, and reduced serum ALT and AST activities, as well as MDA levels and common inflammatory markers. Histopathological examination revealed a dose-dependent attenuation of liver damage with increasing JHB levels. These findings indicate that JHB treatment may augment the activity of alcohol-metabolizing enzymes within this model, and mediate its protective effects through potential mechanisms linked to antioxidant capacity and the regulation of ethanol metabolism.

## Data Availability

The original contributions presented in the study are included in the article/supplementary material, further inquiries can be directed to the corresponding authors.
